# Fluidized-Bed-Roasted Cocoa Has Different Chemical Characteristics than Conventionally Roasted Cocoa

**DOI:** 10.1021/acs.jafc.3c01678

**Published:** 2023-06-23

**Authors:** Ruth Fabiola Peña-Correa, Burçe Ataç Mogol, Christos Fryganas, Vincenzo Fogliano

**Affiliations:** † Department of Food Quality and Design, Wageningen University & Research, P.O. Box 8129, 6700 EV Wageningen, The Netherlands; ‡ Department of Food Engineering, Food Quality and Safety (FoQuS) Research Group, Hacettepe University, Beytepe, 06800 Ankara, Turkey

**Keywords:** Theobroma cacao L., reducing sugars, amino acids, polyphenols, acrylamide, 5-hydroxymethylfurfural, melanoidins

## Abstract

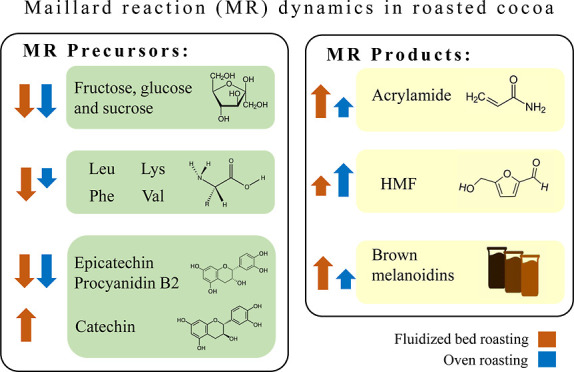

The roasting process can modulate sensory and physicochemical characteristics of cocoa. This study compared the chemical characteristics of cocoa nibs roasted by a convective oven [slow roasting—(SR)] vs cocoa nibs roasted in a fluidized bed roaster [fast roasting—(FR)] at two temperatures (120 and 140 °C). The contents of sugars, free amino acids (FAAs), polyphenols, acrylamide, 5-hydroxymethylfurfural, and melanoidins were monitored. Roasting reduced fructose, glucose, and sucrose contents by 95, 70, and 55%, respectively. The concentration of total FAAs was reduced up to 40% at 140 °C. The FAA profile revealed that FR favored the reactivity of some amino acids (Leu, Lys, Phe, and Val) relevant in the formation of aroma compounds and melanoidins. FR resulted in the generation of more intense brown melanoidins, a significant increase in catechin content, a higher formation of acrylamide, and a lower formation of 5-hydroxymethylfurfural in cocoa compared to SR.

## Introduction

A number of chemical reactions occur during the roasting process of cocoa beans (the fermented and dried seeds of *Theobroma cacao* L.) with a prominent role played by Maillard reaction (MR). In a well-designed manufacturing process, MR mainly leads to improvement in texture,^1^ the generation of chocolate aroma,^2^ and the reduction in bitterness.^3^ Changes in browning can also take place in cocoa due to the generation of melanoidins; however, they are less evident than in coffee due to the natural brown color of unroasted cocoa.^4^

Monosaccharides, FAAs, peptides, proteins, and polyphenols are the main reactants of MR in cocoa.^5^ The complexity of the interactions of these compounds during heat treatments has been thoroughly investigated.^6−14^ The reagents in cocoa beans exposed to the roasting process generate different MR products. Some of the intermediate ones are volatile low molecular weight compounds like Strecker aldehydes and pyrazines,^9^ which are responsible for the pleasant flavor of roasted cocoa.^2^ Acrylamide and 5-hydroxymethylfurfural (HMF) are non-volatile intermediate MR products. However, HMF may also be formed via sugar caramelization reactions. HMF and acrylamide, especially the latter, have been considered thermal process contaminants due to their potential adverse effects on humans.^15^

During the roasting process of cocoa, at the advanced stages of MR, melanoidins are formed. It is well known that they are high molecular weight compounds (HMWCs) that exert diverse yellow to brown hues, and are mainly composed of α-dicarbonyl compounds (a carbohydrate-based skeleton) and partially branched by amino compounds.^16^ Unfortunately, their molecular structure is not fully defined yet. Different studies have demonstrated the presence of covalently bound phenolic compounds (e.g., epicatechin and catechin) within cocoa melanoidins^17−20^ and have assessed the antioxidant activity that these compounds certainly confer to this advanced MR product.^10,12^ However, the precise pathway of incorporation of polyphenols to food melanoidins has not been described yet. It is probably associated with condensation reactions occurring during the MR. When polyphenols undergo condensation reactions during heat treatments like roasting, their bitterness and astringency are reduced, thus improving the overall cocoa flavor.^3^

Different roasting conditions can modulate MR by modifying the concentration of the above-mentioned reactants and products. However, relatively little innovation in cocoa roasting has been proposed in the last 50 years.^5^

Fluidized bed roasting is a technique originally developed for coffee processing. The technique is based on blowing a strong flow of hot air from the bottom of the roasting chamber, thus making the solid particles to constantly move, resembling a stirring fluid.^21^ This technique involves almost 100% convective heat transfer and is recognized for its low-carbon footprint.^22^ In contrast, oven roasting is mainly based on conductive heat transfer from the metallic surface of the trays, thus resembling the traditional cocoa roasting. In our previous studies,^4,23^ we found that the heat transfer efficiency of fluidized bed roasting reduced the roasting time by a factor of 12 compared to traditional roasting. As a consequence, fluidized bed roasting led to (i) a higher porosity, thus suggesting a deeper heat penetration within cocoa nibs^23^ and (ii) a lower reduction in water activity,^4^ which favored the formation of nitrogen-heterocyclic compounds such as pyrazines. The changes in the chemical composition that fluidized bed roasting causes to the precursors and the MR products in cocoa deserve investigation.

The objective of the current study is to compare the effect of two different roasting techniques (fluidized bed roasting and convective oven roasting with aluminum trays) and two temperatures (120 and 140 °C) on the concentration of typical MR reactants (i.e., sugars, amino acids, and polyphenols) and MR products (HMF, acrylamide, and melanoidins) in cocoa nibs.

## Materials and Methods

### Chemicals and Samples

Fermented and dried Forastero cocoa beans (*T. cacao* L.) from Ivory Coast, with a water content of 6.14 ± 0.02% w/w, were supplied by Olam International (Koog aan de Zaan, The Netherlands). Petroleum ether with a boiling point between 40 and 60 °C, methanol (99.9%), and acetonitrile (HPLC grade) were purchased from Actu-All Chemicals (Oss, The Netherlands). Formic acid (99%) was purchased from Sigma-Aldrich (St. Louis, USA). Carrez I (potassium hexacyanoferrate(II) trihydrate 10.6% m/v) and Carrez II (zinc sulfate 22% m/v) solutions were purchased from Chem-Lab (Zedelgem, Belgium). Cellulose acetate (CA) and polytetrafluoroethylene (PTFE) syringe filters were purchased from Phenomenex (Utrecht, The Netherlands). Oasis HLB solid phase extraction cartridges were purchased from Waters (Milford, MA, USA). The following standards were obtained in analytical standard quality (purity ≥97%) from Sigma-Aldrich (St. Louis, USA): d-(−)-fructose, d-(+)-glucose, sucrose, l-alanine, l-arginine, l-asparagine, l-aspartic acid, l-glutamine, l-glutamic acid, l-glycine, l-histidine, l-isoleucine, l-leucine, l-lysine, l-methionine, l-phenylalanine, l-proline, l-serine, l-threonine, l-tryptophan, l-tyrosine, l-valine, (−)-epicatechin, (+)-catechin, procyanidin B2, chlorogenic acid, gallic acid, caffeic acid, ferulic acid, acrylamide, and 5-hydroxymethylfurfural. The system of PureLab Ultra (ELGA LabWater, Lane End, UK) prepared MilliQ water.

### Roasting Process of Cocoa Nibs

Fermented and dried cocoa beans (180 g) were heated in a pre-heated pan using an induction stove (ATAG BV, The Netherlands) with constant stirring (4 min) and then cooled (40 °C). The shells were manually removed and winnowed with an air gun. The cotyledons (i.e., the cocoa nibs) were softly cracked with a mortar and pestle and then sieved to retain the cocoa particles between 4.0 and 7.0 mm. The sieved cocoa nibs (water content of 4.31 ± 0.11% w/w) were kept in vacuum bags at −20 °C until the roasting experiments.

The roasting process of sieved cocoa nibs in a fluidized bed roaster and a convective oven was performed as described in our previous study.^23^ Briefly, samples of cocoa nibs (70 g) were roasted in a pre-heated electric fluidized bed coffee roaster (Toper Optical Roaster, Izmir, Turkey) at 120 and 140 °C for 6 min and 27 s, and 3 min and 43 s, respectively. This study refers to this technique as fast roasting or FR. For the oven roasting procedure, batches of 70 g of cocoa nibs were dispersed on aluminum trays and then placed in a pre-heated electric convective oven (VWR International B.V., Breda, The Netherlands) at 120 °C for 73 min and 18 s, and at 140 °C for 45 min and 16 s. This procedure was referred to as slow roasting or SR in this study. The roasting times were determined by a water content of 1.0 ± 0.1% w/w in roasted cocoa nibs. The two roasting techniques and the two roasting temperatures resulted in four different roasting conditions abbreviated as FR-120 (i.e., FR/roasted at 120 °C), FR-140 (i.e., FR/roasted at 140 °C), SR-120 (i.e., SR/roasted at 120 °C), and SR-140 (i.e., SR/roasted at 140 °C). Unroasted (UR) cocoa nibs (200 g) were separated as a control. All the samples were vacuum-packed and stored at −20 °C until usage.

### Preparation of Cocoa Powder

A defatting procedure with organic solvent was performed to obtain cocoa powder (CP) (defatted ground cocoa nibs), as described in our previous study.^23^ Briefly, cocoa nibs were twice ground using a screw juicer (Vital Max Oscar 900, Hurom, Korea). Ground cocoa (100 g) was defatted with petroleum ether (4 × 200 mL). After each defatting step, the mix was stirred (15 min, RT) and then allowed to precipitate for 1 h. The upper layers containing the solvent with the cocoa butter were pipetted off, pooled, and centrifuged (2950*g*, 10 min, RT). The solvent containing cocoa butter was discarded, and the pellets and the sediments (CP) were air-dried (48 h, RT) in a fume hood, while they were gently stirred with a spatula every 12 h. The fat-free and solvent-free cocoa samples, namely, CP, were put into airtight plastic containers and stored at −20 °C until they were used for chemical analysis.

### Measurement of pH in Cocoa Samples

Solutions of 0.1 g CP/mL were prepared in MilliQ water. They were vortexed (1 min, RT) and centrifuged (3220*g*, 5 min, RT). The supernatants were used to measure the pH with a calibrated digital pH-meter (Serial Nr. 11110295, UWR International, Germany).

### Determination of Free Sugar Content in Cocoa Powders

Fructose, glucose, and sucrose were determined on 2.5 g of CP extracted with 10.5 mL of MilliQ water, 12.5 mL of ethanol, and 1 mL of each Carrez solution I and II. First, the mixture was heated in a water bath (60 min, 50 °C) and centrifuged (8960*g*, 5 min, 5430 R, Eppendorf, Hamburg, Germany). The supernatant was filtered through a 0.2 μm CA syringe filter (28 mm diameter) and collected into HPLC vials. The extraction process was performed in duplicate. The samples were immediately analyzed using an HPLC Ultimate 3000 system 1 (Thermo Fisher Scientific, Walthman, MA USA) with a RI-501 detector (Shodex, Munchen, Germany) and an evaporative light scattering detector (Polymer Labs, Washington, US) under these settings: injection volume 20 μL, evaporation temperature 90 °C, nebulizer temperature 50 °C, carrier flow 1.60 mL/min; a carbohydrate ES column (5 μm, 250 mm × ID 4,6 mm, Prevail) was used. The elution was achieved using 75% acetonitrile and 25% Milli-Q water. Calibration curves of fructose, glucose, and sucrose (0.25–2.00 mM) were prepared with their respective standards. Finally, the results were processed using the software Chromeleon version 7.2.6 (Thermo Fisher Scientific, Walthman, MA USA). The limits of detection and quantification for fructose, glucose, and sucrose, calculated from the signal noise area, were 0.01 and 0.03 mM, respectively.

### Determination of FAA Content in Cocoa Powders

For the determination of the FAAs alanine (Ala), arginine (Arg), asparagine (Asn), aspartic acid (Asp), glutamine (Gln), glutamic acid (Glu), glycine (Gly), histidine (His), isoleucine (Ile), leucine (Leu), lysine (Lys), methionine (Met), phenylalanine (Phe), proline (Pro), serine (Ser), threonine (Thr), tryptophan (Trp), tyrosine (Tyr), and valine (Val) in CP, 1 g of the sample was extracted three times with 20 mL of water (10–5–5 mL) and vortexed (5 min) in each stage. After centrifugation (4650*g* × 3 min), the supernatants were collected and combined. A sample of the combined extract (200 μL) was mixed with acetonitrile (1:4, v/v), centrifuged (4650*g* × 3 min), and filtered through a 0.45 μm nylon syringe filter into an HPLC vial. The FAAs were analyzed using an Agilent Ultivo system (Santa Clara, CA USA) consisting of an Agilent 1260 Infinity II LC system coupled to an Agilent 6465 triple quadrupole mass spectrometer according to the method described by Salman and co-workers (2021).^24^ The extraction was performed in duplicate.

### Determination of Polyphenols in Cocoa Powders

The flavan-3-ols epicatechin, catechin, and Procyanidin B2 (P-B2) and the phenolic acids chlorogenic acid, gallic acid, caffeic acid, and ferulic acid were determined on 100 mg of CP. The sample was vortexed with 5 mL of MilliQ water and 5 mL of methanol (10 min) and centrifuged (12.902*g*, 10 min, RT). The supernatant was filtrated through 0.2 μm PTFE filters (15 mm diameter) and collected into HPLC amber vials.

The extracts were analyzed by using a Nexera UPLC system (Shimadzu Corporation, Kyoto, Japan) coupled with a LCMS-8050 triple quadrupole mass spectrometer (Shimadzu Corporation, Kyoto, Japan). The UPLC unit consisted of a SIL-30AC autosampler, a LC-20ADXR solvent delivery module, a DGU-20ASR degassing unit, a CTO-20AC column oven, and a FCV-20AH_2_ valve unit. 5 μL of each sample contained in vials were injected into a Kinetex Evo C18 column (2.6 μm, 2.1 × 100 mm, 100 Å, Phenomenex, Torrance, CA, USA). The flow rate was set at 0.5 mL/min, and the column temperature at 40 °C. The mobile phases consisted of 0.2% formic acid (solvent A), acetonitrile with 0.2% formic acid (solvent B) with the following elution profile (*t* in [min]/[% B]): (0.0/5), (0.5/5), (2.0/25), (5.0/50), (7.0/95), (8.5/95), (8.60/5), and (12.5/5). The voltage of the turbo ion-spray ionization was 4.0 kV. The temperature of the electrospray ionization probe, desolvation line, and heat block was set at 300, 250, and 400 °C, respectively. The pressure of the gas for the collision-induced dissociation was 4 kPa, whereas the flow rates of the drying gas, nebulizer gas, and heating gas were set at 10, 3, and 10 mL/min, respectively. The electrode voltage of Q1 pre bias (collision cell energy entrance potential), collision cell Q2 (collision energy), Q3 pre bias (collision cell energy exit potential), and parent and fragment ion *m*/*z* of the multiple reaction monitoring transitions were optimized using standard solutions of the target analytes (concentration 10–20 mg/L) and the support software (Shimadzu Corporation, Kyoto, Japan). The dwell time and the time window for MS data acquisition in the negative mode were also optimized for single reaction monitoring (SRM). The most abundant fragment ion was selected for quantitation. The second and third fragments in ion yield were selected for structural confirmation based on the optimized SRM transition (Table S1). Finally, the data were processed with the software LabSolutions (Shimadzu Corporation, Kyoto, Japan). The concentration of each phenolic compound in samples was calculated by means of a calibration curve built in the range between 25 μg/L and 15 mg/L. The limits of detection and quantification, based on the standard deviation of the blank response, can be observed in Table S1.

### Determination of Acrylamide and 5-HMF in Cocoa Powders

An extraction procedure was carried out in duplicate for the quantification of acrylamide and HMF in 1 g of CP. The cocoa sample was added with 9 mL of 10 mM formic acid, 0.5 mL of Carrez I, and 0.5 mL of Carrez II solutions. The mixture was vortexed (3 min) and centrifuged (2.950*g*, 5 min). The pellet was re-extracted twice with 5 mL of 10 mM formic acid. All supernatants were combined, then centrifuged (8.960*g*, 5 min, RT), and finally kept at −20 °C until analysis.

For acrylamide analysis, 1 mL of the supernatant was passed through a preconditioned (first 1 mL methanol then 1 mL water) Oasis MCX solid phase extraction cartridge, and the pure extract was analyzed using the LC–MS/MS according to the method described by Žilić and co-workers (2020).^25^ The limits of detection and quantification of acrylamide, calculated from the signal noise area, were 3 and 10 μg/kg cocoa, respectively.

For HMF analysis, 1 mL of supernatant was passed through a preconditioned (first 1 mL methanol then 1 mL water) HLB solid-phase extraction cartridge. The eluent was discarded, and HMF was eluted with 1 mL of methanol and collected into HPLC glass vials for further HPLC analysis in an HPLC Ultimate 3000 Dionex (Thermo Fisher Scientific, Walthman, MA USA) equipped with a Diode Array Detector and a temperature-controlled column oven. The chromatographic separations were performed on a C18-A 4.6 × 150 mm 5 μm Polaris column (Agilent, Santa Clara, CA USA) using a mixture of 10 mM aqueous formic acid solution and acetonitrile (95:5, v/v) at a flow rate of 1.0 mL/min for 28 min at 20 °C. Data acquisition was performed by recording chromatograms at 283.5 nm using the software Chromeleon 7.2 (Thermo Fisher Scientific, Walthman, MA USA). The concentration of HMF was calculated through a calibration curve built in the range between 0.1 and 10 μg/mL. The limits of detection and quantification of HMF, calculated from the signal noise area, were 0.03 and 0.10 μg/mL, respectively.

### Determination of Water-Soluble Compounds above 20 kDa

To obtain native high molecular-weight compounds and high molecular-weight melanoidins, an extractive procedure was performed based on a previous study.^18^ Briefly, 10 g of CP was thoroughly mixed with 80 mL of Milli-Q water using a homogenizer (4 min, 9000 rpm, ULTRA-TURRAX, T25 digital, Probe T4, IKA, Staufen, Germany). The mixture was capped and placed in a shaking hot water bath (70 °C, 20 min, 80 rpm) and centrifuged (22.680*g*, 15 min, Avanti ultra-centrifuge, Rotor ID 16250, Beckman Coulter, USA). The pellet was discarded, and the supernatant was successively vacuum-filtered through Whatman filter papers Nr 4, 44, and 602.

The last filtrate was ultrafiltered in a stirring cell unit (Amicon, model 8400, max volume 400 mL, Millipore, Billerica, USA) equipped with a 20 kDa membrane (Mycrodyn Nadir, Nadir FM UP020Pes, Sterlitech, USA) under a positive pressure of 4.5 bar generated by nitrogen supply. Three washing steps with 30 mL of Milli-Q water were done on the retentate when its volume was about 20 mL. The filtrates were discarded, and the retentate (about 20 mL) was freeze-dried at −80 °C (Christ, Alpha 2–4 LDplus, Osterode am Harz, Germany). The weight of the dry fraction (*W*_HMWC_), and the weight of the CP (*W*_CP_) on a dry basis (d.b.) were used to calculate the relative content of water-soluble HMWC > 20 kDa of CP, according to eq 1.
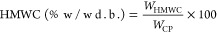
1

### Analysis of Brown Compounds

Solutions of 3.3 mg/mL were prepared with the freeze-dried HMWC extracted from each cocoa sample and filtered with 0.45 nm CA filters. The browning intensity was determined by measuring their absorbance (420 nm, RT) with a spectrophotometer (Cary 50 UV–vis spectrophotometer, Varian, Australia). MilliQ water was used as a blank.

### Statistical Analysis

Data were statistically analyzed by ANOVA. The least significant difference (LSD) method with 95% significance was applied using the statistic software StatGraphics Centurion XVIII (StatGraphics Technologies Inc., USA). The heat map analysis with dendrogram clustering was performed using R commander 3.6.1 (R Foundation, Austria) and R studio (RStudio Team, USA).

## Results and Discussion

### pH in Cocoa

The pH in unroasted and roasted cocoa was measured to confirm their acidity and to know to what extent the pH was affected by roasting conditions. The pH of unroasted cocoa was 5.44 ± 0.13, and it was significantly different from the four roasting conditions (*p* < 0.05), which ranged from 5.29 to 5.32 without significant differences among them (*p* > 0.05). The effect of roasting on decreasing the pH of CP was also reported.^18^ Non-enzymatic browning reaction in model systems also demonstrated a gradual decrease in pH.^26^

The fact that pH is not different among the roasted samples is relevant because the differences between the roasting conditions we will report in the coming paragraphs cannot be attributed to the pH.

It is well known that the Maillard reaction is dependent on the pH of the food. At low pH (<7), the formation of furanic compounds (e.g., HMF) from Amadori rearrangement products is favored, whereas the routes to reductones and fission products, which are mainly responsible for the formation of volatile aromatic compounds, are preferred at pH > 7.^27^ In addition, the non-enzymatic browning reactions are accelerated under neutral or alkaline conditions.^26^

### Decrease in Sugar Content in Roasted Cocoa

**Figure 1:**
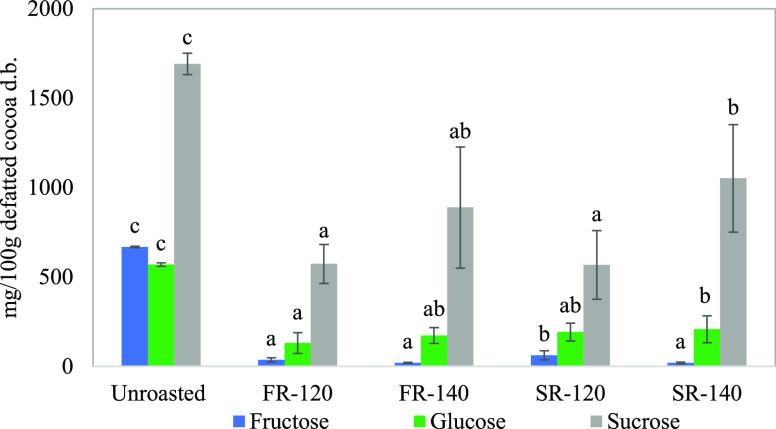
Fructose, glucose, and sucrose contents in unroasted cocoa and in cocoa roasted under different conditions: fast roasting at 120 °C (FR-120) and 140 °C (FR-140), and slow roasting at 120 °C (SR-120) and 140 °C (SR-140). The results are expressed in mg/g of CP d.b. The error bars correspond to the standard deviations and the lowercase letters represent significant differences (*p* < 0.05) among roasting conditions for each kind of sugar.

Fructose, glucose, and sucrose contents in unroasted CP were 667.59 ± 4.25, 569.56 ± 9.38, and 1691.26 ± 59.74 mg/100 g d.b., respectively. These values were significantly reduced (*p* < 0.05) during the four roasting conditions, as shown in Figure 1. The decreases in fructose (by approximately 95%) and glucose (by approximately 70%) were more pronounced than in sucrose (by approximately 55%). Free sugar reduction was expected as MR occurs during roasting. Also, the order of reactivity is logical, as fructose and glucose are reducing sugars that directly react with an amine source, while sucrose first needs to be hydrolyzed into its monomers.^28^

Fructose content in SR-120 cocoa was significantly higher (*p* < 0.05) than that in cocoa roasted under the other three roasting conditions, which were not significantly different (*p* > 0.05) (Figure 1). There was a significant effect of roasting temperature on fructose content only by SR, with the highest reduction at the highest temperature. There was no significant effect of roasting temperature on glucose content (*p* > 0.05); however, FR cocoa had the lowest glucose content compared to SR cocoa at the same roasting temperatures, suggesting a slight influence of the roasting technique (*p* > 0.05). Regarding the sucrose content, it was not significantly affected by the roasting techniques (*p* > 0.05), but it was influenced by roasting temperature under SR (*p* < 0.05) and FR (*p* > 0.05) conditions: the lower the temperature, the higher the reduction of sucrose. For each roasting technique, this inverse effect of roasting temperature in sucrose reduction is likely due to the time effect: the experiments performed at 120 °C required about 40% extra time to reach the same water content as those performed at 140 °C, as reported in the section “Roasting Process of Cocoa Nibs”. Thus, our data could suggest that the conversion of sucrose into its monomers might be more time-dependent than temperature-dependent.

Our results of fructose, glucose, and sucrose contents in cocoa cannot be easily compared with previous studies, as the content of sugars in cocoa beans is affected by heterogeneous conditions of fermentation.^29^ Despite the differences in sugar contents, the general effect of roasting in the reduction of fructose, glucose, and sucrose contents presented in this study is aligned with other studies.^29,30^

### Reduction of FAA Content in Roasted Cocoa

**Figure 2:**
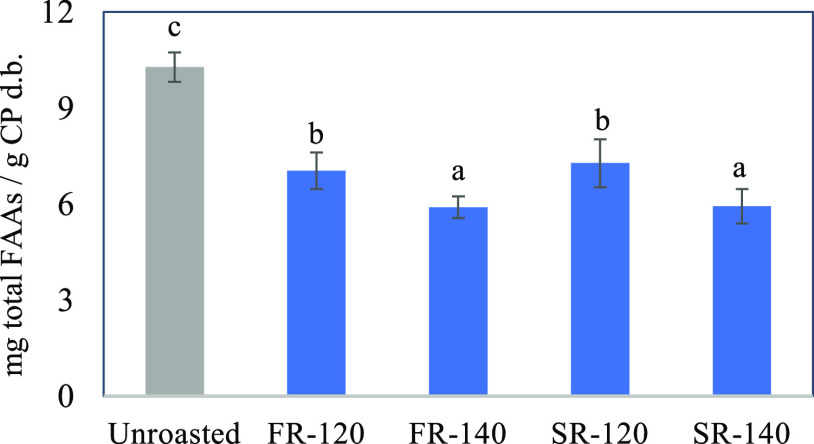
Total FAA content in unroasted cocoa and cocoa roasted under different conditions: Fast roasting at 120 °C (FR-120) and 140 °C (FR-140), and slow roasting at 120 °C (SR-120) and 140 °C (SR-140). The results are expressed in mg/g of CP d.b. The error bars correspond to the standard deviations and the lowercase letters represent significant differences (*p* < 0.05).

During heat treatments, two opposite phenomena influence the FAA concentration in cocoa: amino acids are released via degradation of proteins and peptides,^31^ and FAA are consumed to produce volatile odor-active compounds^1,32^ and melanoidins.^33^ The single FAA content in cocoa for each roasting condition and control is presented in Table S2, and the sum of them is shown in Figure 2. We found a significant reduction (*p* < 0.05) in total FAA content in cocoa under the four conditions. Likely, the consumption of FAAs via MR was predominant over the release of FAAs. The different roasting techniques had no significant effect on FAA content, but the higher roasting temperature resulted in a higher decrease (40% at 140 °C to 30% at 120 °C). These phenomena can be associated with the temperature dependence of formation of volatile compounds such as pyrazines^4^ and melanoidins.^34^

Comparing the content of FAAs in cocoa with other studies is far from simple. Similar to sugar content, FAAs are greatly affected by fermentation,^35^ which may not be the same for the cocoa samples used in various studies. Apart from this, many studies only covered a part of the 20 common amino acids, making it difficult to compare the total FAA content of cocoa.^32,35−39^ However, the reduction of total FAA upon roasting is aligned with other studies.^32,36−39^

**Figure 3:**
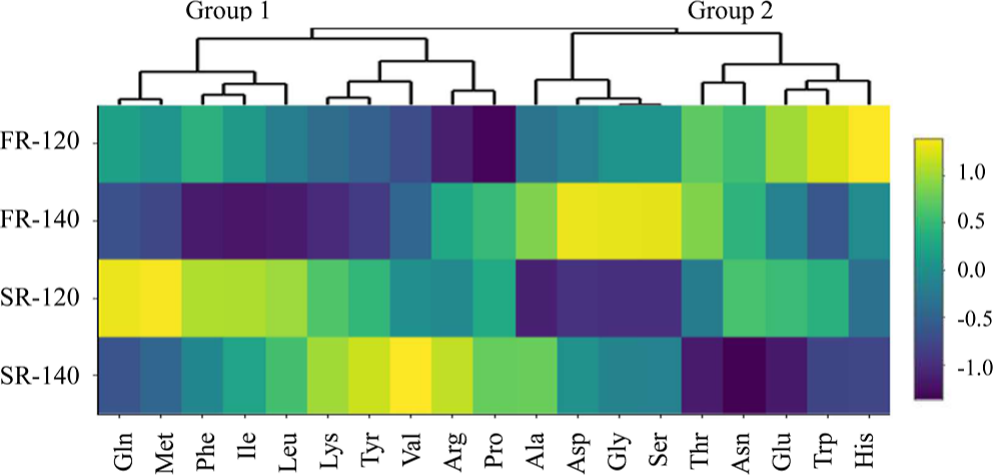
Heatmap of the relative abundance of FAA content (based on mmol) in CP (d.b.) obtained from cocoa nibs roasted under different conditions: fast roasting at 120 °C (FR-120) and 140 °C (FR-140), and slow roasting at 120 °C (SR-120) and 140 °C (SR-140). The data were scaled per column, with zero being the mean with a standard deviation of 1. Data were accompanied by color: the mean is represented by green color, values above the mean are represented by yellow color, and values below the mean are represented by deep purple color.

The content of each amino acid decreased under the four roasting conditions (Table S2); however, there was a higher decrease of some amino acids under specific roasting treatments, as reflected in the heatmap presented in Figure 3. The heatmap was generated by converting the data of Table S2 to mmol/g CP d.b. and then normalizing the new data. The vertical dendrogram of the heatmap separates two main groups of amino acids: group 1 consists of the amino acids Gln, Met, Phe, Ile, Leu, Lys, Tyr, Val, Arg, and Pro, and group 2 Ala, Asp, Gly, Ser, Thr, Asn, Glu, Trp, and His. The roasting technique determined this grouping: FR cocoa had the lowest content of FAA in group 1, while SR cocoa had the lowest content of FAA in group 2, as observed in the purple areas in Figure 3. The roasting temperature determined the subdivision of group 2 with SR-120 having the lowest content of Ala, Asp, Gly, and Ser, and SR-140 having the lowest content of Thr, Asn, Glu, Trp, and His. Group 1 is also subdivided into two groups; however, the separation was not determined by roasting temperature.

The MR chemistry is influenced by the degree of specificity of the amino acids to generate products like volatile organic compounds. According to the cocoa-model systems performed by Arnoldi and co-workers (1988),^9^ Leu, Lys, Phe, Val, Thr, Glu, Ala, and Asp are amino acids involved in generating diverse pleasant odor-active volatile compounds via MR like pyrazines and Strecker aldehydes. Their results reported that Leu can produce 11 pyrazines out of 22, followed by Thr with 9, and Val and Phe with 8 each. Ala, Asp, and Glu contributed to the formation of 4, 3, and 2 pyrazines, respectively.^9^ Cha and co-workers (2019)^40^ confirmed Leu’s ability to produce various pyrazine types.

The ability of Leu to produce more pyrazine types than other amino acids is exceptional. It was the second most abundant amino acid in our unroasted cocoa (Table S2), and fluidized bed roasting, specially FR-140, showed the lowest content of it. Phe and Val were the fourth and the sixth most abundant FAAs (Table S2), and their consumption was also favored by FR. In other words, the balance between the release and the degradation of Leu, Phe, and Val was in favor of the degradation under the fluidized bed technique, probably toward the formation of MR products such as volatile organic compounds. This hypothesis is supported by our previous studies,^4,23^ which reported higher formation of pyrazines by the fluidized bed technique than by traditional oven roasting.

Other factors like pH and water activity have essential roles in the performance of MR.^8,41^ As mentioned above, the pH did not significantly change under the four roasting conditions. However, the roasting techniques affected the *a*_w_ of cocoa.^4^ In one of our previous studies, we found that the *a*_w_ of cocoa during the second half of the FR process fluctuated between 0.25 and 0.45 (unpublished data) with a final *a*_w_ ≈ 0.30,^4^ while the *a*_w_ during the second half of the SR process ranged between 0.15 and 0.30 (unpublished data), with a final *a*_w_ ≈ 0.20.^4^ This difference might have also determined the FAA profile.

In summary, these results demonstrated that the reactivity of amino acids can be modulated by the roasting technique, and that the fluidized bed technique was more suitable to reduce the relative content of key MR amino acids (i.e., Leu, Phe, and Val) than oven roasting, probably toward the formation of MR products.

### Changes in Polyphenol Content in Roasted Cocoa

**Figure 4:**
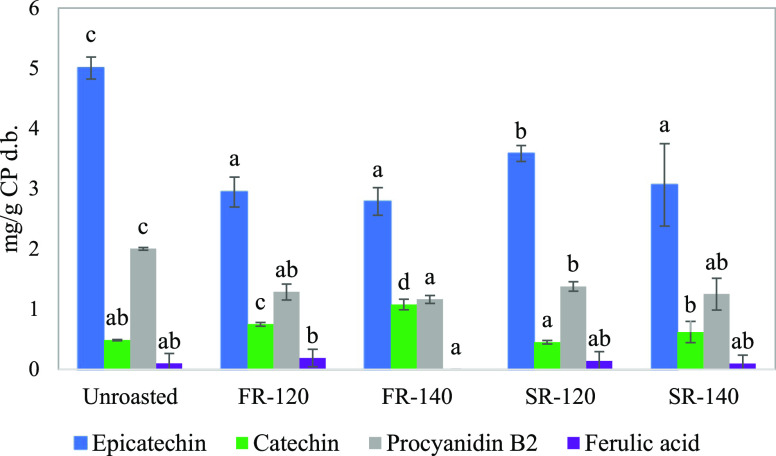
Epicatechin, catechin, procyanidin B2, and ferulic acid contents (mg/g CP d.b.) obtained from unroasted cocoa nibs, and cocoa nibs roasted under different conditions: fast roasting at 120 °C (FR-120), fast roasting at 140 °C (FR-140), slow roasting at 120 °C (SR-120), and slow roasting at 140 °C (SR-140). The error bars correspond to the standard deviations, and the lowercase letters represent significant differences (*p* < 0.05) among roasting conditions for each phenolic compound.

The changes in epicatechin, catechin, procyanidin B2 (P-B2), and ferulic acid content in cocoa under the four roasting conditions are presented in Figure 4. Chlorogenic acid, caffeic acid, and gallic acid were also analyzed, as they have been found in cocoa;^17^ however, we did not detect them in our samples.

The effect of the four roasting conditions on the content of epicatechin, catechin, P-B2, and ferulic acid is presented in Figure 4. As expected, the four roasting processes significantly reduced (*p* < 0.05) the two most abundant phenolic compounds in cocoa, i.e., epicatechin and P-B2. Epicatechin might have undergone epimerization and condensation reactions, and P-B2 depolymerization and condensation reactions.^42^ SR-120 proved to be the least severe treatment with the lowest reduction in epicatechin and P-B2, and no significant difference in catechin content (*p* > 0.05). Catechin content increased in cocoa under SR-140 (*p* > 0.05), FR-120 (*p* < 0.05), and FR-140 (*p* < 0.05) conditions, and was also determined by roasting temperature: the higher the temperature (140 °C), the higher the catechin content. Catechin content was also influenced by the roasting technique (*p* < 0.05): at equal roasting temperatures, fluidized bed-roasted cocoa had the highest catechin content. The increase in catechin content could be due to the epimerization reactions of epicatechin that regularly occur during heating treatments,^42^ which seem to be favored by FR. Depolymerization reactions of structures containing catechin, e.g., procyanidins B1, B3, and B4,^42^ could have also contributed to catechin increase. Lastly, the concentration of ferulic acid in cocoa was not significantly affected by any roasting condition.

The sum of epicatechin, catechin, P-B2, and ferulic acid contents in unroasted, FR-120, FR-140, SR-120, and SR-140 cocoa, accounted for 7.59 ± 0.30, 5.17 ± 0.45, 5.03 ± 0.36, 5.55 ± 0.35, and 5.02 ± 1.46 mg/g CP d.b, respectively. The four roasting conditions significantly reduced the sum of these compounds (*p* < 0.05). SR-120 had the highest total polyphenol content of the four kinds of roasted cocoa; however, there were no statistical differences among the four treatments (*p* > 0.05). Similar results on epicatechin, P-B2, and catechin contents in unroasted cocoa, and the effect of roasting on the decrease of epicatechin and P-B2 have been reported.^30,43−46^ Regarding the dynamics of catechin content during roasting, there are some discrepancies among studies: Żyżelewicz and co-workers (2016)^46^ demonstrated that catechin increases steadily during the first 15 min of roasting (135 and 150 °C) and then gradually drops to its initial content. According to De Taeye and co-workers (2017),^43^ the balance between epicatechin epimerization and catechin degradation was in favor of a catechin increase in criollo cocoa beans but not in Forastero and Trinitario, which showed a decrease in catechin content. In contrast, Oracz and Nebesny (2019)^30^ reported a similar trend in catechin decrease for the same three cocoa varieties.

The results presented in this study demonstrate that roasting leads to a decrease in epicatechin and P-B2 contents in cocoa, and that the increase in catechin content, probably via epimerization reactions of epicatechin, is more profound underfluidized bed roasting.

### Formation of Acrylamide and HMF in Roasted Cocoa

The formation of acrylamide and HMF was monitored in order to assess the impact of the different roasting conditions on the formation of processing contaminants, as shown in Table 1.

**Table 1:** Acrylamide and HMF Contents in CP Obtained from Cocoa Nibs Roasted under Different Conditions*

Roasting condition	μg Acrylamide/kg CP d.b.	mg HMF/kg CP d.b.
Unroasted	370.58 ± 12.90^a^	not detected
FR-120	2031.56 ± 23.88^c^	3.56 ± 0.06^a^
FR-140	1992.13 ± 113.75^c^	4.43 ± 0.34^b^
SR-120	1552.36 ± 288.95^b^	12.48 ± 0.22^c^
SR-140	1335.64 ± 202.43^b^	18.01 ± 0.14^d^

* The results are expressed as means ± standard deviations. Lowercase letters in the same column represent significant differences (*p* < 0.05).

Acrylamide was detected in unroasted cocoa nibs (Table 1). This compound is produced during the fermentation^47^ and drying processes^48^ of cocoa in the field. In addition, the limited thermal load involved during the deshelling process may have contributed.

Acrylamide content in cocoa significantly increased under the four roasting conditions (*p* < 0.05), especially FR-120 and FR-140, which led to a higher concentration of this compound than the SR process at the same roasting temperatures (*p* < 0.05). The roasting temperature had no effect on the generation of this thermal contaminant, as presented in Table 1. Farah and co-workers (2012)^49^ found a higher content of acrylamide in cocoa samples (2200 to 4000 μg/kg d.b. of defatted cocoa) than in our study. In contrast, the study of Żyżelewicz et al. (2016)^50^ reported a much lower formation of acrylamide (100 to 500 μg/kg d.b. of defatted cocoa). The effect of roasting temperature was reported in both studies; however, they found different trends. Farah et al. (2012)^49^ observed a temperature increase followed by a rise in the acrylamide content at a constant roasting time. On the contrary, Żyżelewicz et al. (2016)^50^ detected the highest acrylamide content in cocoa beans that were roasted for the longest time but at the lowest of the tested temperatures. These discrepancies may be ascribed to differences in the experimental design, cocoa bean varieties, thermal load, extractive procedure, and analytical method. According to Desmarchelier et al. (2022),^51^ cocoa is one of the most challenging food matrices for acrylamide analysis due to interferences.

The routes of generation of acrylamide have been extensively studied, the Maillard reaction being one of the most common mechanisms of formation, in which free asparagine is an essential precursor.^52,53^ As expected, the absolute content of asparagine was significantly reduced under the four roasting conditions (*p* < 0.05, Table S2). However, the trends did not directly correlate with acrylamide formation: the absolute content of asparagine in cocoa was significantly affected by roasting temperature (*p* < 0.05) but not by the roasting technique (*p* > 0.05). Diverse chemical reactions that occur in food matrixes interfere with consuming reactants and forming products. For example, the biogenic amine of asparagine, 3-aminopropionamide, was reported to be more likely to produce acrylamide than free asparagine during cocoa roasting.^47^

HMF was not detected in unroasted cocoa despite the thermal load of the drying and deshelling processes (Table 1). The four roasting conditions significantly increased HMF content in cocoa (*p* < 0.05). The roasting techniques determined HMF formation, with SR cocoa having a significantly higher HMF content than FR cocoa (*p* < 0.05). Table 1 also shows that the higher the roasting temperature, the higher the HMF content, with SR having the most profound effect. Two aspects of FR cocoa could have mitigated the formation of HMF: (i) HMF concentration strongly increases with the decreasing *a*_w_.^54^ As elaborated above, fluidized bed roasting led to a lower reduction in *a*_w_ in cocoa nibs than oven roasting.^4^ (ii) The higher content of free catechin and other phenolic compounds present in FR cocoa could have trapped carbonyl compounds, thus hindering the formation of HMF.^11,55^

Our study demonstrated that, although unroasted cocoa had a pH < 7 and contained the reactants to produce HMF either via sugar dehydration or MR,^15^ the formation of this compound was mitigated when using a fluidized bed roaster, even at 140 °C which is a higher temperature within the regular range for cocoa roasting.^5^ HMF content in our roasted cocoa was comparable with the results reported by Quiroz-Reyes and Fogliano (2018)^18^ (17–75 mg/kg CP), while Sacchetti (2016)^34^ reported lower values (0.1–0.8 mg/kg CP) and Maldonado-Mateus et al. (2021)^56^ reported much higher values (3000 to 8000 mg/kg CP). Several factors (e.g., the pH, the chemical composition of the raw material, and the roasting procedure) could have accounted for these differences. Moreover, various chemical reactions in food matrices may simultaneously occur. For example, HMF being a carbonyl compound can be involved in MR to produce other MR products, such as acrylamide.^57^ Despite the variation in HMF content in roasted cocoa, these studies and ours agree on the same trend: HMF content increases as roasting temperature increases.

The balance between the formation and degradation of acrylamide and HMF during roasting might determine their final concentration in cocoa. Our study demonstrated that FR favored the formation of acrylamide, while SR favored the formation of HMF.

### Amount of Water-Soluble HMWCs and Their Brown Color Intensity

Cocoa melanoidins are brown-colored heterogeneous HMWCs roughly ranging from 30 to 70 kDa.^30^ The content of water-soluble HMWCs (>20 kDa) in unroasted and roasted cocoa is presented in Table 2. Data show that soluble HMWCs (e.g., polysaccharides, proteins, and condensed polyphenols) constitute 3% of unroasted CP (d.b.). The content of HMWCs in FR-120 and FR-140 cocoa was slightly lower (*p* > 0.05) compared to that in unroasted cocoa. In contrast, HMWC content in SR was significantly higher (*p* < 0.05) than that in unroasted cocoa: an increase of 50 and 100% was observed in SR-140 and SR-120 CP, respectively.

**Table 2:** Content and Absorbance of Water Soluble HMWCs of Cocoa Roasted under Different Conditions*

Roasting condition	g HMWC/100 g CP d.b.	Absorbance of HMWC solutions (3.3 mg/mL) at 420 nm
Unroasted	2.99 ± 0.05^a^	0.42 ± 0.00^a^
FR-120	2.80 ± 0.66^a^	0.55 ± 0.02^bc^
FR-140	2.80 ± 0.04^a^	0.63 ± 0.07^d^
SR-120	6.52 ± 0.33^c^	0.51 ± 0.06^b^
SR-140	4.43 ± 1.16^b^	0.62 ± 0.04^cd^

* The data correspond to means ± standard deviations. Lowercase letters in the same column represent significant differences (*p* < 0.05).

These data suggest that during roasting, native HMWCs could have depolymerized to low molecular weight compounds. At the same time, brown melanoidins were generated via condensation reactions of reducing sugars, FAAs, and polyphenols.^5^ Unfortunately, the ratio of the native HMWC and newly formed melanoidins is unknown, and we can notice melanoidin formation via the increase of browning of the high molecular weight fraction. Quiroz-Reyes and Fogliano (2018)^18^ also found a significant increase in compounds >20 kDa in whole cocoa beans roasted over metallic trays inside a convective oven. Their values were higher than the ones presented in our study: their unroasted CP had about 7% HMWCs (w/w, d.b.), and roasted cocoa up to 17.2%. The lack of fermentation of their cocoa samples is probably responsible for such a difference. Oracz and Nebesny (2019)^30^ did not find significant changes in the content of HMWCs in oven roasted cocoa beans (>12.4 kDa). Even they reported some minor reductions of HMWCs under specific roasting treatments, as we found in FR cocoa. However, the HMWC yield reported in their study (12.5 to 15.0% of HMWCs w/w, d.b) was higher than that in our investigation. This difference may be assigned to the lower cut-off of their membrane, which would have retained more HMWCs.

Although the amount of HMWCs did not significantly change upon FR, data in Table 2 show that the absorbance at 420 nm of FR-HMWCs was significantly higher than that of unroasted cocoa (*p* < 0.05), thus confirming the formation of brown cocoa melanoidins. The brown color intensity of SR-HMWCs was also significantly higher than that of unroasted cocoa (*p* < 0.05). Interestingly, at equal roasting temperatures, FR led to a higher absorbance of the HMWC extracts than SR (*p* > 0.05). The roasting temperature led to significant differences (*p* < 0.05) in both roasting techniques: the temperature increase led to higher brown color intensity. These results support the temperature-dependency of formation of cocoa melanoidins previously reported.^34^

The brown color intensity is not only determined by the concentration of the melanoidins but also by their composition. The type of amino acids involved in the formation of melanoidins determines their brownness, as demonstrated in various model system experiments.^8,10,58^Table 3 shows that Lys is known for its intense brown color formation. Ala, Gly, Ile, Leu, Met, Phe, Ser, Trp, Tyr, and Val are also capable of generating intense to medium brown colors. In contrast, the amino acids Arg, Asn, Asp, Cys, Gln, Glu, His, Pro, and Thr have been classified as medium to low brown color producers.

**Table 3:** Amino Acids with the Highest Capacity of Formation of Brown Melanoidins^a^

Amino acid	Brown color intensity	
	High	Medium
Ala	z	x
Gly	x, z	
Ile	z	x
Leu	z	x, y
Lys	x, y, z	
Met	z	x
Ser	z	x
Trp	x	
Tyr	x, z	
Val	z	x

a The letters indicate the source of information: (x) Ashoor and Zent (1984),^8^ (y) Lamberts et al. (2008),^58^ and (z) Wong et al. (2008).^10^

By comparing Table 3 with Figure 3, interesting considerations can be made. Fluidized bed-roasted cocoa, especially FR-140, utilized 7 of the 11 high-medium brown producing amino acids (i.e., Ile, Leu, Lys, Met, Phe, Tyr, and Val), while SR favored the reduction of the other four (i.e., Asp, Glu, His, and Thr). This observation suggests that the differences in brownness among the four kinds of HMWCs could have been determined by the type of amino acids that were used to build up melanoidins. Fluidized bed roasting caused the decrease of more high-medium brown-producing amino acids than SR, forming darker melanoidins.

In conclusion, this investigation demonstrated that fluidized bed roasting produces more intense brown melanoidins than conventional roasting. This phenomenon is associated with the type of amino acids probably involved in the formation of melanoidins, as fluidized bed roasting favored the reduction of more high-medium brown-producing amino acids than conventional roasting.

In general, our study provides evidence about modulating MR during the roasting process of cocoa nibs. The possibility to change the sensory, chemical, and physical characteristics of cocoa by changing the roasting conditions offers the opportunity to design different cocoa-based ingredients for diverse final products.

## Supporting Information Available

Single reaction monitoring conditions of the phenolic compounds and absolute content of the free amino acids (PDF)

## Notes

This research falls outside human or animal studies, so institutional ethical approval was not required.

## Abbreviations

*a*_w_: water activity
CP: cocoa powder
d.b.: dry basis
FR: fast roasting/roasted
FR-120: fast roasting/roasted at 120 °C
FR-140: fast roasting/roasted at 140 °C
FAA: free amino acid
HMF: 5-hydroxymethylfurfural
HMWC: high molecular weight compound
MR: Maillard reaction
PB-2: procyanidin B2
RT: room temperature
SRM: single reaction monitoring
SR: slow roasting/roasted
SR-120: slow roasting/roasted at 120 °C
SR-140: slow roasting/roasted at 140 °C
