# Supplementary material for: Fluidized-Bed-Roasted Cocoa Has Different Chemical Characteristics than Conventionally Roasted Cocoa
Source: J Agric Food Chem. 2023 Jun 23;71(26):10203–11. doi: 10.1021/acs.jafc.3c01678 (PMC10326883; doi:10.1021/acs.jafc.3c01678)
Supplement: Supplementary file 1 — jf3c01678_si_002.pdf [file jf3c01678_si_002.pdf]

SUPPLEMENTARY MATERIAL

FLUIDIZED BED ROASTED COCOA HAS DIFFERENT CHEMICAL CHARACTERISTICS THAN CONVENTIONAL ROASTED COCOA

Ruth Fabiola Peña-Correa[a], Burçe Ataç Mogol[b], Christos Fryganas[a], and Vincenzo Fogliano[a*]

[*]Corresponding author: vincenzo.fogliano@wur.nl

[a] Wageningen University & Research, Department of Food Quality and Design, PO Box 8129, 6700 EV Wageningen, The Netherlands.

[b] Hacettepe University, Department of Food Engineering, Food Quality and Safety (FoQuS) Research Group, 06800 Beytepe, Ankara, Turkey

Table S1. Single Reaction Monitoring (SRM) conditions of the phenolic compounds in negative mode determined by LC-MS/MS, and their limits of detection (LOD) and quantification (LOQ).

Compound	Precursor ion (m/z)	Product ion (m/z)	Dwell time (ms)	Q1 Pre Bias (V)	Collision energy (V)	Q3 Pre Bias (V)	LOD (µg/L)	LOQ (µg/L)
Epicatechin	289.1	245.05	4	15	15	12	8.1	24.6
		109.00	4	21	27	11		
		122.85	4	15	31	24		
		202.95	4	15	19	14		
Catechin	289.1	244.95	4	15	15	17	2.3	7.0
		109.00	4	15	25	11		
		122.90	4	15	30	13		
		203.00	4	15	19	14		
Procyanidin B2	577.1	407.10	4	30	24	15	2.9	8.9
		288.95	4	30	26	22		
		424.95	4	30	17	16		
		125.10	4	30	36	13		
Chlorogenic acid	353.1	191.00	4	26	20	13	0.6	1.9
		85.00	4	18	42	13		
		93.05	4	25	42	10		
		126.95	4	26	36	25		
Gallic acid	169.1	125.05	13	20	16	13	2.7	8.2
		78.95	13	10	22	13		
		80.95	13	11	18	13		
		124.55	13	19	25	12		
Caffeic acid	179.1	135.05	5	20	17	14	4.8	14.5
		134.60	5	20	25	13		
		107.05	5	20	22	11		
		88.95	5	21	32	14		
Ferulic acid	193.2	133.95	11	12	17	14	3.2	9.7
		178.00	11	28	15	12		

Table S2. Free amino acids content (mg/g cocoa powder d.b.) in unroasted cocoa and cocoa roasted under different conditions: Fast roasting at 120 °C (FR-120) and 140 °C (FR-140), and slow roasting at 120 °C (SR-120) and 140 °C (SR-140).

Amino Acid	Unroasted	FR-120	FR-140	SR-120	SR-140
Ala	0.89 ± 0.04^{c}	0.78 ± 0.02^{b}	0.71 ± 0.04^{a}	0.76 ± 0.05^{ab}	0.71 ± 0.04^{a}
Arg	0.69 ± 0.08^{c}	0.47 ± 0.01^{ab}	0.46 ± 0.02^{a}	0.54 ± 0.05^{b}	0.49 ± 0.04^{ab}
Asn	1.10 ± 0.05^{c}	0.81 ± 0.09^{b}	0.68 ± 0.03^{a}	0.83 ± 0.09^{b}	0.62 ± 0.08^{a}
Asp	0.35 ± 0.05^{b}	0.32 ± 0.02^{ab}	0.32 ± 0.01^{ab}	0.30 ± 0.03^{a}	0.28 ± 0.01^{a}
Gln	0.08 ± 0.0^{d}	0.01 ± 0.00^{b}	0.00 ± 0.00^{a}	0.01 ± 0.00^{c}	0.00 ± 0.00^{a}
Glu	0.71 ± 0.03^{d}	0.38 ± 0.05^{c}	0.25 ± 0.01^{b}	0.35 ± 0.06^{c}	0.18 ± 0.04^{a}
Gly	0.12 ± 0.00^{c}	0.11 ± 0.00^{b}	0.10 ± 0.00^{b}	0.10 ± 0.01^{ab}	0.09 ± 0.00^{a}
His	0.20 ± 0.01^{c}	0.17 ± 0.06^{bc}	0.12 ± 0.02^{ab}	0.14 ± 0.03^{ab}	0.10 ± 0.02^{a}
Ile	0.46 ± 0.03^{c}	0.37 ± 0.02^{b}	0.30 ± 0.01^{a}	0.40 ± 0.03^{b}	0.32 ± 0.02^{a}
Leu	1.19 ± 0.06^{d}	0.68 ± 0.05^{bc}	0.53 ± 0.03^{a}	0.76 ± 0.06^{c}	0.61 ± 0.06^{ab}
Lys	0.44 ± 0.03^{d}	0.29 ± 0.02^{bc}	0.24 ± 0.01^{a}	0.31 ± 0.03^{c}	0.26 ± 0.02^{ab}
Met	0.04 ± 0.00^{b}	0.00 ± 0.00^{a}	0.00 ± 0.00^{a}	0.00 ± 0.00^{a}	0.00 ± 0.00^{a}
Phe	1.06 ± 0.01^{c}	0.64 ± 0.06^{b}	0.46 ± 0.02^{a}	0.69 ± 0.06^{b}	0.51 ± 0.05^{a}
Pro	0.29 ± 0.01^{c}	0.23 ± 0.01^{ab}	0.21 ± 0.01^{a}	0.26 ± 0.02^{b}	0.21 ± 0.01^{a}
Ser	0.37 ± 0.03^{c}	0.29 ± 0.01^{b}	0.27 ± 0.01^{ab}	0.28 ± 0.02^{b}	0.24 ± 0.01^{a}
Thr	0.32 ± 0.02^{c}	0.23 ± 0.01^{b}	0.20 ± 0.01^{a}	0.23 ± 0.02^{b}	0.18 ± 0.01^{a}
Trp	0.13 ± 0.01^{c}	0.07 ± 0.00^{b}	0.05 ± 0.00^{a}	0.07 ± 0.00^{b}	0.05 ± 0.00^{a}
Tyr	1.09 ± 0.18^{b}	0.56 ± 0.03^{a}	0.47 ± 0.02^{a}	0.61 ± 0.06^{a}	0.52 ± 0.03^{a}
Val	0.65 ± 0.01^{c}	0.52 ± 0.03^{b}	0.44 ± 0.02^{a}	0.54 ± 0.04^{b}	0.46 ± 0.03^{a}
Total	10.27 ± 0.46^{c}	7.03 ± 0.56^{b}	5.89 ± 0.34^{a}	7.27 ± 0.75^{b}	5.92 ± 0.54^{a}

The results are expressed as means ± standard deviations. Lowercase letters in the same row represent significant differences ($p < 0.05$).
